# Disentangling Fatigue Dimensions in Multiple Sclerosis: Differential Associations with Health-Related Quality of Life Outcomes in RRMS

**DOI:** 10.3390/medsci14030357

**Published:** 2026-06-29

**Authors:** Edoardo Sessa, Giovanni Restuccia, Carla Susinna, Gabriele Triolo, Roberta Lombardo, Lilla Bonanno, Concetta Pastura, Angelo Quartarone, Viviana Lo Buono

**Affiliations:** IRCCS Centro Neurolesi Bonino-Pulejo, SS113 Via Palermo C. da Casazza, 98124 Messina, Italy; edoardo.sessa@irccsme.it (E.S.); carla.susinna@irccsme.it (C.S.); gabriele.triolo@irccsme.it (G.T.); roberta.lombardo@irccsme.it (R.L.); lilla.bonanno@irccsme.it (L.B.); concetta.pastura@irccsme.it (C.P.); angelo.quartarone@irccsme.it (A.Q.); viviana.lobuono@irccsme.it (V.L.B.)

**Keywords:** multiple sclerosis, fatigue, health-related quality of life, SF-36, modified fatigue impact scale, depression, relapsing-remitting multiple sclerosis

## Abstract

**Background**: Fatigue is a highly disabling symptom in multiple sclerosis and is strongly associated with reduced health-related quality of life (HRQoL). However, whether distinct fatigue dimensions show differential associations with specific HRQoL domains remains unclear. This study investigated the relationship between physical, cognitive, and psychosocial fatigue and SF-36 outcomes in people with relapsing-remitting multiple sclerosis (RRMS). **Methods**: Forty-four people with RRMS were included in this cross-sectional study. Participants were classified as fatigued or non-fatigued according to the Modified Fatigue Impact Scale (MFIS) total score cut-off. Group differences in SF-36 domains were examined using ANCOVAs adjusted for age, sex, disease duration, and disability, with additional sensitivity analyses adjusting for depressive symptoms. Partial Spearman correlations assessed associations between MFIS subscales and SF-36 domains across the whole sample, controlling for demographic and clinical covariates. **Results**: Eighteen participants were classified as fatigued and 26 as non-fatigued. Fatigued participants showed significantly lower scores across all SF-36 domains. After additional adjustment for depressive symptoms, differences remained significant for Physical Functioning, Role Physical, General Health, Vitality, Social Functioning, and Role Emotional. Physical fatigue was inversely associated with several HRQoL domains, including physical, social, vitality, general health, and mental health-related outcomes. Psychosocial fatigue was associated with poorer Physical Functioning, Role Physical, Bodily Pain, and Social Functioning. Cognitive fatigue was not significantly associated with any SF-36 domain. **Conclusions**: Physical and psychosocial fatigue appear to be the main fatigue dimensions associated with HRQoL impairment in RRMS. Dimension-specific fatigue assessment may help identify more individualized targets for patient-centered management.

## 1. Introduction

Multiple sclerosis (MS) is a chronic immune-mediated disorder of the central nervous system characterized by inflammation, demyelination, and neurodegeneration, resulting in a highly heterogeneous clinical course and a broad spectrum of neurological and non-neurological manifestations. Beyond overt physical disability, people with MS (PwMS) frequently experience so-called “invisible” symptoms that substantially contribute to disease burden and daily functional impairment. Among these, fatigue represents one of the most prevalent and disabling symptoms across all disease stages [1,2]. Although prevalence estimates vary depending on definitions and assessment methods, fatigue affects up to 75% of people with MS during the disease course and is often reported as one of the most disabling symptoms, sometimes exceeding pain and physical disability in terms of perceived impact. Thus, fatigue has been consistently associated with reduced quality of life, diminished social participation, and impaired occupational functioning [3,4].

Despite its clinical relevance, fatigue remains difficult to characterize because of its subjective, heterogeneous, and multifactorial nature. Several disease-related and contextual mechanisms may contribute to fatigue, including inflammatory and neurodegenerative processes, reduced neural efficiency, disability, sleep disturbances, depressive symptoms, pain, medication effects, and reduced physical activity [5,6,7]. Importantly, fatigue is increasingly recognized as a multidimensional construct rather than a unitary symptom. The Modified Fatigue Impact Scale (MFIS), derived from the Fatigue Impact Scale, allows the impact of fatigue to be evaluated across physical, cognitive, and psychosocial domains [1,8]. This distinction is clinically relevant, as physical fatigue may primarily reflect reduced endurance and increased perceived effort during daily activities, cognitive fatigue may involve mental effort, attentional endurance, and processing efficiency, whereas psychosocial fatigue may capture the impact of fatigue on social participation and role engagement.

A growing body of evidence indicates that fatigue is strongly associated with health-related quality of life (HRQoL) in MS. Previous studies have shown that higher fatigue levels are related to poorer physical, emotional, and social functioning, and that fatigue may represent one of the major determinants of HRQoL together with disability and self-efficacy [9,10,11,12]. However, most available studies have focused on global fatigue severity or overall HRQoL, with less attention to whether specific fatigue dimensions are differentially associated with distinct HRQoL domains. This issue is particularly relevant because different components of fatigue may not exert the same impact on daily functioning. For instance, previous evidence suggests that motor and cognitive fatigue may contribute differently to quality of life outcomes in MS [13]. Therefore, disentangling the relationship between fatigue dimensions and HRQoL domains may provide a more clinically informative characterization of fatigue burden.

Moreover, fatigue overlaps with other clinically relevant factors, particularly neurological disability and depressive symptoms. Although these dimensions are interrelated, fatigue cannot be fully reduced to disability or mood disturbance [7]. Controlling for these variables is therefore essential to better isolate the specific contribution of fatigue to HRQoL impairment. Against this background, the present study aimed to investigate the relationship between fatigue and HRQoL in patients with relapsing-remitting MS (RRMS). First, we compared fatigued and non-fatigued patients across the domains of the Short Form-36 Health Survey (SF-36) to identify the HRQoL dimensions most affected by fatigue. Second, we examined whether the physical, cognitive, and psychosocial components of fatigue, as measured by the MFIS, were differentially associated with specific SF-36 domains after accounting for relevant demographic and clinical variables. By moving beyond global fatigue scores, this study seeks to provide a more granular characterization of fatigue-related burden in RRMS and to identify domain-specific patterns that may inform targeted clinical assessment and individualized interventions.

## 2. Materials and Methods

### 2.1. Study Design and Participants

This cross-sectional observational study consecutively included 44 adult subjects with a diagnosis of RRMS according to the 2017 McDonald criteria [14]. Participants were recruited during routine outpatient visits from the MS clinic of the IRCCS Centro Neurolesi Bonino-Pulejo (Messina, Italy) from September 2024 to May 2025. Eligible participants were between 18 and 60 years, had a confirmed diagnosis of RRMS for at least 12 months prior to enrolment with mild-to-moderate disability (EDSS ≤ 4.5) and the ability to complete the clinical and self-report assessments. Participants were excluded if they did not meet the 2017 McDonald criteria for RRMS, had any other neurological disorder (e.g., stroke, epilepsy, neurodegenerative disease, major head trauma with loss of consciousness, etc.), or had an EDSS score > 4.5. Furthermore, PwMS with clinically relevant depressive symptoms were excluded to minimize the potential confounding effect of clinically significant depression on fatigue perception and HRQoL outcomes. Clinically relevant depressive symptoms were defined according to the BDI cut-off score of 14.

Fatigue severity was assessed using the Modified Fatigue Impact Scale (MFIS). Based on the pre-specified MFIS total score cutoff of 38 [15], participants were classified as fatigued or non-fatigued.

The study protocol was reviewed and approved by the Ethics Committee of IRCCS Centro Neurolesi Bonino Pulejo (n. E40/23, 7 February 2023) and conducted in accordance with the ethical principles of the Declaration of Helsinki. All subjects provided written informed consent before participation. This study is reported in accordance with the Strengthening the Reporting of Observational Studies in Epidemiology (STROBE) statement for cross-sectional studies [16].

### 2.2. Clinical Assessment

A comprehensive clinical assessment was conducted by the MS clinic multidisciplinary team to collect the demographic and clinical data suitable for the study. EDSS was administered by a board-certified neurologist during the neurological examination. A trained neuropsychologist evaluated global cognitive functioning using the Montreal Cognitive Assessment (MoCA) and the presence of depressive symptoms with the BDI. Fatigue was assessed by the trained neuropsychologist using the Modified Fatigue Impact Scale (MFIS), a self-report questionnaire derived from the Fatigue Impact Scale [1] and specifically designed to evaluate the impact of fatigue on physical, cognitive and psychosocial functioning [8]. Health-related quality of life (HRQoL) was also assessed by the trained neuropsychologist using the 36-Item Short Form Health Survey (SF-36), a self-report questionnaire comprising 36 items across eight domains: physical functioning, social functioning, role limitations due to physical problems, role limitations due to emotional problems, bodily pain, vitality, mental health, and general health perception. Scores range from 0 to 100, with higher scores indicating better HRQoL. Raw scores were successively adjusted using specific Italian normative data, as necessary.

### 2.3. Statistical Analysis

Distributional assumptions for preliminary between-group comparisons were assessed using the Shapiro–Wilk test. Depending on variable distribution, group differences in demographic and clinical characteristics were assessed using either independent-samples Student’s *t*-tests or Mann–Whitney U tests. Differences in sex distribution were examined using the chi-square test.

To investigate group differences in HRQoL, separate analyses of covariance (ANCOVAs) were performed for each SF-36 domain. In the primary models, group (fatigued vs. non-fatigued) was entered as the fixed factor, while age, sex, disease duration, and EDSS were included as covariates. Effect sizes were reported as omega squared (ω^2^). Sensitivity analyses were then performed by repeating the ANCOVA models with additional adjustment for BDI.

To further characterize fatigue severity at a dimensional level, associations between MFIS total score and selected clinical variables were examined across the whole RRMS sample using Spearman’s or partial Spearman’s correlation analyses, depending on the variable of interest. In addition, partial Spearman correlation analyses were performed across the whole RRMS sample to examine the associations between the three MFIS subscales (Physical, Cognitive, and Psychosocial) and the SF-36 domains. These analyses were adjusted for age, sex, disease duration, EDSS, and BDI total score. To account for multiple testing in the exploratory correlation analyses between MFIS subscales and SF-36 outcomes, *p*-values were adjusted using the Benjamini–Hochberg false discovery rate (FDR) correction. FDR correction was applied across the family of MFIS subscale × SF-36 outcome correlations. By contrast, *p*-values reported for ANCOVA models are uncorrected, as these analyses were considered planned comparisons. Statistical significance was set at *p* < 0.05. Statistical analyses were performed using JASP (0.95.4.0, https://jasp-stats.org/).

## 3. Results

### 3.1. Sample Characteristics

The sample characteristics are summarized in Table 1. A total of 44 RRMS subjects were included, of whom 26 were classified as non-fatigued and 18 as fatigued. The two groups did not differ significantly in age (39.85 ± 9.99 vs. 36.78 ± 9.04 years, *p* = 0.416), sex distribution (female sex: 88.5% vs. 83.3%, *p* = 0.626), education (14.12 ± 3.43 vs. 13.06 ± 3.37 years, *p* = 0.392), disease duration (9.62 ± 6.72 vs. 8.67 ± 6.95 years, *p* = 0.607), or EDSS (3.02 ± 0.73 vs. 3.28 ± 0.62, *p* = 0.227). By contrast, the fatigued group showed lower MoCA scores (24.14 ± 2.65 vs. 25.94 ± 2.40, *p* = 0.023) and higher BDI scores (6.17 ± 4.02 vs. 1.23 ± 1.14, *p* < 0.001). Subjects in the fatigued group also showed markedly higher MFIS scores across all subscales (*p* < 0.001), including Physical (25.00 ± 5.52 vs. 12.92 ± 7.31), Cognitive (22.39 ± 6.57 vs. 7.96 ± 4.15), Psychosocial (5.28 ± 1.84 vs. 1.89 ± 1.40), and Total scores (52.67 ± 11.03 vs. 22.77 ± 10.53).

### 3.2. Group Differences in Health-Related Quality of Life

**Figure 1:**
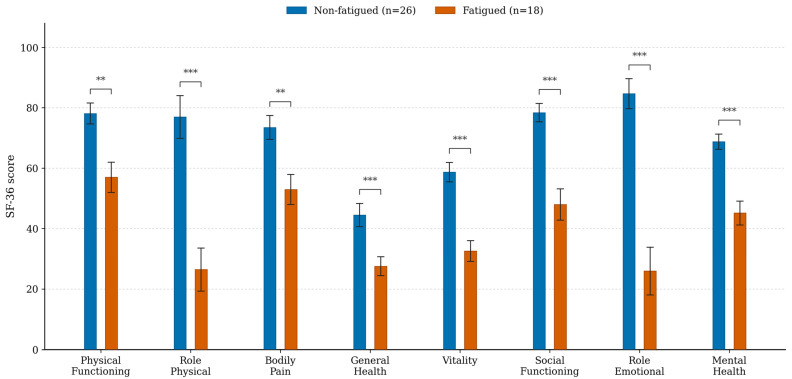
Differential SF-36 profiles according to fatigue status. Grouped bar plot showing mean SF-36 domain scores in non-fatigued and fatigued people with RRMS. Error bars represent SEM. Higher scores indicate better health-related quality of life. Group differences were assessed using ANCOVA adjusted for age, sex, disease duration, and EDSS. ** *p* < 0.01; *** *p* < 0.001. Additional sensitivity ANCOVAs adjusted for BDI score were further conducted. Several group differences in HRQoL remained significant: specifically, the fatigued group continued to show lower scores in Physical Functioning, Role Physical, General Health, Vitality, Social Functioning, and Role Emotional. By contrast, group differences were no longer significant for Bodily Pain and Mental Health. A comparison of primary and BDI-adjusted ANCOVAs results is reported in Table 3.

Fatigued pwMS showed lower scores in Physical Functioning (F(1,38) = 11.85, *p* = 0.001, ω^2^ = 0.170), Role Physical (F(1,38) = 21.55, *p* < 0.001, ω^2^ = 0.323), Bodily Pain (F(1,38) = 8.86, *p* = 0.005, ω^2^ = 0.155), General Health (F(1,38) = 15.35, *p* < 0.001, ω^2^ = 0.210), Vitality (F(1,38) = 25.02, *p* < 0.001, ω^2^ = 0.343), Social Functioning (F(1,38) = 22.90, *p* < 0.001, ω^2^ = 0.326), Role Emotional (F(1,38) = 35.02, *p* < 0.001, ω^2^ = 0.432), and Mental Health (F(1,38) = 23.97, *p* < 0.001, ω^2^ = 0.348) (Table 2 and Figure 1).

**Table 3:** Comparison of primary and BDI-adjusted ANCOVA models.

SF-36 Domain	*p* Without BDI	ω^2^ Without BDI	*p* with BDI	ω^2^ with BDI
Physical Functioning	0.001 **	0.170	0.018 *	0.090
Role Physical	<0.001 ***	0.323	0.029 *	0.082
Bodily Pain	0.005 **	0.155	0.286	0.004
General Health	<0.001 ***	0.210	0.019 *	0.086
Vitality	<0.001 ***	0.343	0.013 *	0.108
Social Functioning	<0.001 ***	0.326	0.007 **	0.140
Role Emotional	<0.001 ***	0.432	0.012 *	0.092
Mental Health	<0.001 ***	0.348	0.184	0.012

* *p* < 0.05; ** *p* < 0.01; *** *p* < 0.001.

### 3.3. Correlations Between Fatigue and Clinical Variables

Exploratory analyses were conducted across the whole RRMS sample, showing that the MFIS total score was not significantly associated with age (ρ = 0.029, *p* = 0.852). Similarly, no significant association emerged between MFIS total score and disease duration after adjusting for age and sex (ρ = −0.025, *p* = 0.875). By contrast, MFIS total score showed a significant positive association with EDSS after adjustment for age, sex, and disease duration (ρ = 0.384, *p* = 0.013). Furthermore, MFIS total score was not significantly associated with MoCA performance after adjustment for sex and disease duration (ρ = −0.270, *p* = 0.084). Conversely, the MFIS total score was positively associated with the BDI score after adjustment for age, sex, disease duration, and EDSS (ρ = 0.688, *p* < 0.001).

### 3.4. Correlations Between Fatigue and Quality of Life

Higher MFIS Physical scores were significantly associated with lower Physical Functioning (ρ = −0.627, *p* < 0.001), Role Physical (ρ = −0.476, *p* = 0.007), General Health (ρ = −0.519, *p* = 0.005), Vitality (ρ = −0.554, *p* = 0.002), Social Functioning (ρ = −0.490, *p* = 0.007), and Mental Health (ρ = −0.392, *p* = 0.038).

The MFIS Psychosocial subscale also showed significant inverse associations with several QoL dimensions, including Physical Functioning (ρ = −0.576, *p* = 0.002), Role Physical (ρ = −0.513, *p* = 0.005), Bodily Pain (ρ = −0.472, *p* = 0.007), and Social Functioning (ρ = −0.421, *p* = 0.024).

By contrast, the MFIS Cognitive subscale was not significantly associated with any SF-36 domain. No significant association was observed between any MFIS subscale and the Health Transition item. Results are also reported in Table 4.

## 4. Discussion

The present study provides evidence that fatigue in RRMS is a multidimensional and clinically heterogeneous construct with non-uniform associations with health-related quality of life (HRQoL). By combining group-level comparisons and dimension-specific analyses, our findings move beyond global fatigue severity and suggest that different fatigue components contribute differently to patient-perceived disease burden. At the group level, fatigued patients showed significantly lower SF-36 scores across all domains, despite being comparable to non-fatigued patients in age, sex, education, disease duration, and neurological disability. This pattern is consistent with previous evidence showing that fatigue is among the most important determinants of HRQoL in MS, together with disability and self-efficacy, and that its burden extends across physical, emotional, and social domains [9,10,11,12]. Importantly, in the present study, HRQoL impairment was observed in a sample with mild-to-moderate disability, suggesting that fatigue captures a clinically meaningful dimension of RRMS burden that is not fully reflected by conventional disability measures.

A relevant finding concerns the role of depressive symptoms. Although patients with clinically relevant depressive symptoms were excluded, the fatigued group still showed higher BDI scores than the non-fatigued group. These residual differences reflect subclinical variations in depressive symptoms rather than clinically significant depression, which was excluded by the study criteria. Additional adjustment for BDI attenuated several between-group differences, and the associations with Bodily Pain and Mental Health were no longer significant. This suggests that depressive symptoms partly contribute to fatigue-related HRQoL impairment, especially in domains more closely related to affective well-being and pain perception. This interpretation is consistent with previous models identifying depression, sleep disturbance, and disease severity as relevant contributors to fatigue in MS [5,7,8]. However, group differences remained significant for Physical Functioning, Role Physical, General Health, Vitality, Social Functioning, and Role Emotional, supporting the view that fatigue is not reducible to depressive symptomatology. Rather, fatigue and depressive symptoms appear to be partially overlapping but non-redundant constructs, possibly reflecting shared affective, behavioral, and disease-related mechanisms.

The dimensional analyses further clarified this relationship. Physical fatigue emerged as the most consistent correlate of reduced HRQoL, showing significant inverse associations with Physical Functioning, Role Physical, General Health, Vitality, Social Functioning, and Mental Health. This widespread pattern suggests that the physical component of fatigue is not limited to motor exhaustion or reduced endurance, but may influence several aspects of daily functioning, including perceived health, social participation, and emotional well-being. Previous studies have similarly emphasized the strong contribution of fatigue to quality of life and daily functioning in MS, with some evidence suggesting that motor or physical fatigue may have particularly relevant effects on HRQoL domains [12,13,17]. In patients with mild-to-moderate disability, higher physical fatigue scores were associated with greater functional limitations, higher perceived effort during daily activities, and reduced participation, independently of neurological disability level.

From a mechanistic perspective, this interpretation is compatible with models suggesting that MS-related fatigue reflects increased perceived effort, reduced neural efficiency, and altered regulation of motor and cognitive resources [5,18]. Neurophysiological and neuroimaging studies have linked fatigue to impaired conduction, energy failure, altered thalamocortical connectivity, and white matter abnormalities, supporting the view that fatigue may arise from both structural and functional disruption within distributed neural systems [19,20,21,22]. However, because the present study did not include neuroimaging or objective fatigability measures, these mechanisms should be considered as explanatory frameworks rather than direct evidence from our dataset.

Higher MFIS Psychosocial scores were related to lower Physical Functioning, Role Physical, Bodily Pain, and Social Functioning. This finding suggests that psychosocial fatigue scores were associated with poorer role engagement, interpersonal functioning, and behavioral participation, consistent with qualitative evidence describing fatigue as a socially limiting experience. Qualitative work has emphasized that MS-related fatigue is often experienced as a disruptive and socially limiting symptom, affecting daily routines, identity, participation, and perceived autonomy [23,24]. In this sense, psychosocial fatigue may represent the subjective and behavioral translation of fatigue burden into reduced participation and social functioning.

Importantly, psychosocial fatigue was the only MFIS subscale significantly associated with Bodily Pain after adjustment for demographic and clinical variables. Although this cross-sectional association does not allow mechanistic conclusions, it may be interpreted within a biopsychosocial framework in which fatigue-related withdrawal, reduced activity, affective load, and maladaptive pain appraisal may be interrelated with pain-related HRQoL impairment within a broader symptom network. Pain is highly prevalent in MS and is closely intertwined with fatigue, mood, and participation restrictions [25,26,27]. Ecological momentary assessment studies also indicate that pain, fatigue, depressive symptoms, and perceived cognitive difficulties fluctuate dynamically in daily life, supporting the idea that these symptoms may interact over time rather than operate as isolated domains [28]. Therefore, the association between psychosocial fatigue and Bodily Pain may reflect a broader symptom network involving pain perception, emotional burden, and reduced behavioral engagement.

In contrast, the MFIS Cognitive subscale was not significantly associated with any SF-36 domain. This finding should be interpreted cautiously and should not be taken to indicate that cognitive fatigue is clinically irrelevant in RRMS. Although this dissociation may partly reflect limited statistical power and restricted variability in symptom burden within our mild-to-moderate RRMS sample, it may also reflect a mismatch between the construct being measured and the outcome instrument used. The SF-36 does not include items targeting attentional endurance, mental effort, slowed information processing, or sustained cognitive performance. By contrast, its physical and role functioning domains, which did show significant associations with physical and psychosocial fatigue, are populated by items referring to everyday physical activities (e.g., walking, climbing stairs, carrying groceries) and role limitations due to physical or emotional problems, constructs that map more directly onto the behavioral and somatic manifestations captured by the MFIS Physical and Psychosocial subscales. Cognitive fatigue, by contrast, primarily affects internally experienced mental effort and processing efficiency during cognitively demanding tasks, dimensions that are not represented in SF-36 item content and that may require MS-specific HRQoL instruments, ecological momentary assessment approaches, or performance-based cognitive tasks designed to capture fatigue-related decline over time. The divergence in findings across fatigue dimensions may therefore reflect not a true absence of clinical impact, but rather the differential sensitivity of the SF-36 to fatigue manifestations that are behaviorally visible versus those that are primarily experienced as increased mental effort and reduced cognitive endurance. This interpretation is consistent with evidence showing that subjective cognitive fatigue may be only weakly associated with objective cognitive performance and may depend on task duration, contextual demands, and sustained mental effort [29,30,31,32]. It is also coherent with the absence of a significant association between MFIS total score and MoCA performance in the present sample, supporting the idea that subjective cognitive fatigue and global cognitive screening measures reflect partially distinct dimensions.

The null association between cognitive fatigue and SF-36 domains may also have clinical implications. Cognitive fatigue may be more strongly related to ecologically complex activities, such as work performance, multitasking, studying, driving, or prolonged cognitive engagement, which are not specifically captured by the SF-36. Cognitive impairment is a well-established contributor to functional limitations in MS, particularly in domains involving information processing speed, memory, and executive functioning [33]. However, the subjective experience of cognitive fatigue may require more specific and dynamic assessment approaches than those offered by generic HRQoL instruments. Future studies should therefore complement the SF-36 with MS-specific HRQoL scales, ecological momentary assessment, work-related functioning measures, and prolonged cognitive tasks designed to capture fatigue-related decline over time.

Altogether, these findings support the need to assess fatigue in RRMS as a multidimensional symptom rather than as a unitary construct. This is particularly relevant because previous work has suggested that fatigue profiles in MS may often be driven more by overall fatigue severity than by clearly separable fatigue dimensions [34]. In contrast, our findings suggest that, when HRQoL domains are considered separately and relevant covariates are controlled, physical and psychosocial fatigue may show distinct patterns of association with patient-reported outcomes. Global fatigue scores may therefore obscure clinically relevant differences between physical, psychosocial, and cognitive fatigue. This has potential implications for intervention planning. Physical fatigue may benefit from strategies targeting endurance, activity pacing, energy conservation, and exercise-based rehabilitation, while psychosocial fatigue may require approaches addressing behavioral activation, emotional regulation, motivation, pain coping strategies, and social participation. Exercise-based and multimodal interventions have shown promise for MS-related fatigue, although tailoring treatment to specific fatigue dimensions may further improve clinical relevance [24,35]. An additional aspect worth considering is the potential relationship between fatigue and progression independent of relapse activity (PIRA), a recently recognized mechanism of disability accumulation in multiple sclerosis that occurs independently of clinical relapses [36]. PIRA is increasingly considered a major driver of long-term disability and is thought to reflect ongoing neurodegenerative processes and compartmentalized inflammation. Within this framework, fatigue may not only represent a secondary consequence of disability or a correlate of inflammatory disease activity but may also be associated with more insidious and subclinical mechanisms of disease progression. Although the cross-sectional design of the present study precludes any causal inference, the observed associations between fatigue severity and functional impairment may be conceptually consistent with the hypothesis that fatigue reflects, at least in part, underlying processes related to PIRA. Longitudinal studies incorporating clinical, imaging, and biological markers are needed to clarify whether fatigue may serve as an early clinical correlate or potential indicator of progression independent of relapse activity.

Several limitations should be acknowledged. First, the relatively small sample size may have limited statistical power, particularly for detecting weaker associations or domain-specific effects. Second, the inclusion of only patients with RRMS and mild-to-moderate disability (EDSS ≤ 4.5) was intended to reduce clinical heterogeneity and improve internal validity; however, this choice restricts the applicability of the findings to patients with secondary or primary progressive MS and to those with more advanced disability. In these populations, fatigue may be more closely intertwined with severe neurological impairment, comorbid conditions, and reduced functional independence, potentially leading to different patterns of association with health-related quality of life. Third, the cross-sectional design prevents conclusions about directionality or causality between fatigue dimensions and HRQoL. Fourth, fatigue, depressive symptoms, and HRQoL were assessed using self-report instruments, which may partly share subjective response components. Fifth, although depressive symptoms were statistically controlled, the overlap between fatigue and mood-related symptoms remains difficult to fully disentangle. Furthermore, the exclusion of participants with clinically relevant depressive symptoms, while methodologically motivated to reduce confounding, may limit the generalizability of the findings to the broader RRMS population, given that fatigue-depression comorbidity has been reported in 50–70% of PwMS. Future studies should include patients across the full spectrum of depressive symptom severity to better capture the heterogeneity of fatigue burden in clinical practice. Finally, potentially relevant contributors to fatigue, such as sleep quality, pain characteristics, physical activity levels, lesion burden, and objective fatigability measures, were not included. Additionally, the SF-36 is a generic HRQoL instrument that does not include items specifically targeting cognitive effort, attentional endurance, or mental fatigue; the absence of significant associations between cognitive fatigue and SF-36 domains should therefore be interpreted as a methodological constraint rather than a definitive indication of clinical irrelevance.

## 5. Conclusions

Despite these limitations, the present study highlights the clinical value of a dimension-specific approach to fatigue in RRMS. Physical and psychosocial fatigue were the main fatigue dimensions associated with poorer HRQoL, whereas cognitive fatigue appeared to follow a more context-dependent pattern that may not be adequately captured by generic HRQoL measures. These findings suggest that more precise phenotyping of fatigue may help identify individualized treatment targets and improve patient-centered management in RRMS.

## Figures and Tables

**Table 1:** Clinical and demographic characteristics of the sample.

	Non-Fatigued (n = 26)	Fatigued (n = 18)	*p*-Value
Demographics			
Age, years	39.85 ± 9.99	36.78 ± 9.04	0.416 ^a^
Female Sex, n (%)	23 (88.5)	15 (83.3)	0.626 ^b^
Education, years	14.12 ± 3.43	13.06 ± 3.37	0.392 ^a^
Clinical measures			
Disease Duration, years	9.62 ± 6.72	8.67 ± 6.95	0.607 ^a^
EDSS, score	3.02 ± 0.73	3.28 ± 0.62	0.227 ^c^
MoCA, score	25.94 ± 2.40	24.14 ± 2.65	0.023 *^c^
BDI, score	1.23 ± 1.14	6.17 ± 4.02	<0.001 ***^a^
Fatigue outcomes			
MFIS Physical, score	12.92 ± 7.31	25.00 ± 5.52	<0.001 ***^c^
MFIS Cognitive, score	7.96 ± 4.15	22.39 ± 6.57	<0.001 ***^c^
MFIS Psychosocial, score	1.89 ± 1.40	5.28 ± 1.84	<0.001 ***^c^
MFIS Total, score	22.77 ± 10.53	52.67 ± 11.03	<0.001 ***^c^

Data are expressed as mean ± SD. ^a^ Mann–Whitney test, ^b^ Chi-square Test, ^c^ Student’s *t*-test. Abbreviations: BDI = Beck Depression Inventory; EDSS = Expanded Disability Status Scale; MFIS = Modified Fatigue Impact Scale; MoCA = Montreal Cognitive Assessment. * *p* < 0.05; *** *p* < 0.001.

**Table 2:** ANCOVA results for SF-36 domains in fatigued versus non-fatigued.

SF-36 Domain	Non-Fatigued (n = 26)	Fatigued (n = 18)	F(1,38)	*p*-Value	ω^2^
Physical Functioning	78.08 ± 17.78	56.94 ± 21.22	11.85	0.001 **	0.170
Role Physical	76.92 ± 36.00	26.39 ± 30.28	21.55	<0.001 ***	0.323
Bodily Pain	73.46 ± 20.15	52.92 ± 21.04	8.86	0.005 **	0.155
General Health	44.42 ± 19.61	27.50 ± 13.20	15.35	<0.001 ***	0.210
Vitality	58.65 ± 16.53	32.50 ± 14.58	25.02	<0.001 ***	0.343
Social Functioning	78.37 ± 15.64	47.92 ± 21.97	22.90	<0.001 ***	0.326
Role Emotional	84.62 ± 25.35	25.93 ± 33.44	35.02	<0.001 ***	0.432
Mental Health	68.77 ± 12.85	45.11 ± 16.79	23.97	<0.001 ***	0.348

Analyses are adjusted for age, sex, disease duration, and EDSS. ** *p* < 0.01; *** *p* < 0.001.

**Table 4:** Associations between fatigue dimensions and SF-36 domains in the overall RRMS sample.

SF-36 Domain	MFIS Physical	MFIS Cognitive	MFIS Psychosocial
Physical Functioning	ρ = −0.627; *p* < 0.001 ***	ρ = −0.274; *p* = 0.128	ρ = −0.576; *p* = 0.002 **
Role Physical	ρ = −0.476; *p* = 0.007 **	ρ = −0.163; *p* = 0.362	ρ = −0.513; *p* = 0.005 **
Bodily Pain	ρ = −0.354; *p* = 0.061	ρ = −0.109; *p* = 0.550	ρ = −0.472; *p* = 0.007 **
General Health	ρ = −0.519; *p* = 0.005 **	ρ = −0.189; *p* = 0.291	ρ = −0.300; *p* = 0.111
Vitality	ρ = −0.554; *p* = 0.002 **	ρ = −0.297; *p* = 0.111	ρ = −0.271; *p* = 0.128
Social Functioning	ρ = −0.490; *p* = 0.007 **	ρ = −0.230; *p* = 0.196	ρ = −0.421; *p* = 0.024 *
Role Emotional	ρ = −0.363; *p* = 0.056	ρ = −0.289; *p* = 0.116	ρ = −0.343; *p* = 0.066
Mental Health	ρ = −0.392; *p* = 0.038 *	ρ = −0.064; *p* = 0.700	ρ = −0.287; *p* = 0.116
Health Transition	ρ = 0.097; *p* = 0.577	ρ = −0.252; *p* = 0.157	ρ = −0.299; *p* = 0.111

Analyses are adjusted for age, sex, disease duration, EDSS, and BDI. Reported *p*-values are Benjamini–Hochberg FDR-adjusted. * *p* < 0.05; ** *p* < 0.01; *** *p* < 0.001.

## Data Availability

The datasets used and analyzed during the current study are available from the corresponding author on reasonable request, because the data are not publicly available due to privacy or ethical restrictions.

## Abbreviations

The following abbreviations are used in this manuscript:

BDI	Beck Depression Inventory
EDSS	Expanded Disability Status Scale
HRQoL	Health-related quality of life
IRCCS	Istituto di Ricovero e Cura a Carattere Scientifico
MFIS	Modified Fatigue Impact Scale
MoCA	Montreal Cognitive Assessment
MS	Multiple sclerosis
PwMS	People with multiple sclerosis
QoL	Quality of life
RRMS	Relapsing-remitting multiple sclerosis
SF-36	36-Item Short Form Health Survey
STROBE	Strengthening the Reporting of Observational Studies in Epidemiology
